# Prosthetic Rehabilitation After Transfemoral Amputation in a Patient With an Ipsilateral Girdlestone Hip: A Case Report

**DOI:** 10.7759/cureus.59175

**Published:** 2024-04-27

**Authors:** Yohei Tanaka, Takaaki Ueno

**Affiliations:** 1 Rehabilitation Medicine, JR Tokyo General Hospital, Tokyo, JPN

**Keywords:** lower limb amputation, transfemoral amputation, girdlestone procedure, prosthetic rehabilitation, transfemoral lower limb prosthesis

## Abstract

To date, there have been no reported cases of patients walking with a prosthesis after receiving an ipsilateral transfemoral amputation following the Girdlestone procedure. We administered a four-month prosthetic rehabilitation program to a 66-year-old man after his transfemoral amputation following the Girdlestone procedure. As a result, he was able to walk using the prosthesis for his daily activities. The prosthesis socket featured a quadrilateral configuration. The patient's ability to ambulate after the Girdlestone procedure was attributed to his ischial tuberosity serving as the primary load-bearing site in the transfemoral prosthesis. With appropriate prosthetic design, fabrication, and rehabilitation, patients can walk using a transfemoral prosthesis even in cases of transfemoral amputation following the Girdlestone procedure.

## Introduction

To date, there are no publications on ipsilateral transfemoral amputation following the Girdlestone procedure and subsequent rehabilitation outcomes. An abstract of a poster presentation reported a case of ipsilateral knee disarticulation following the Girdlestone procedure, but no detailed report is available [1].

The Girdlestone procedure involves the removal of the femoral head from the hip joint. It was developed by the English orthopedic surgeon Gathorne Robert Girdlestone in 1928 as a treatment for septic arthritis [2]. Although 91% of patients reported pain relief postoperatively in a six-year follow-up study [3], achieving adequate functional mobility recovery remains a challenge, with only about 29% walking independently [4] and 28.5% using crutches one year postoperatively [5].

On the other hand, study results indicate 50% gait acquisition with lower limb prostheses for unilateral transtibial amputees and 20% for unilateral transfemoral amputees [6].

It is apparent that the combination of the Girdlestone procedure with an ipsilateral transfemoral amputation makes walking more challenging than either technique alone. This case report is the first to detail the rehabilitation progress of ipsilateral transfemoral amputees following the Girdlestone procedure.

## Case presentation

**Figure 1:**
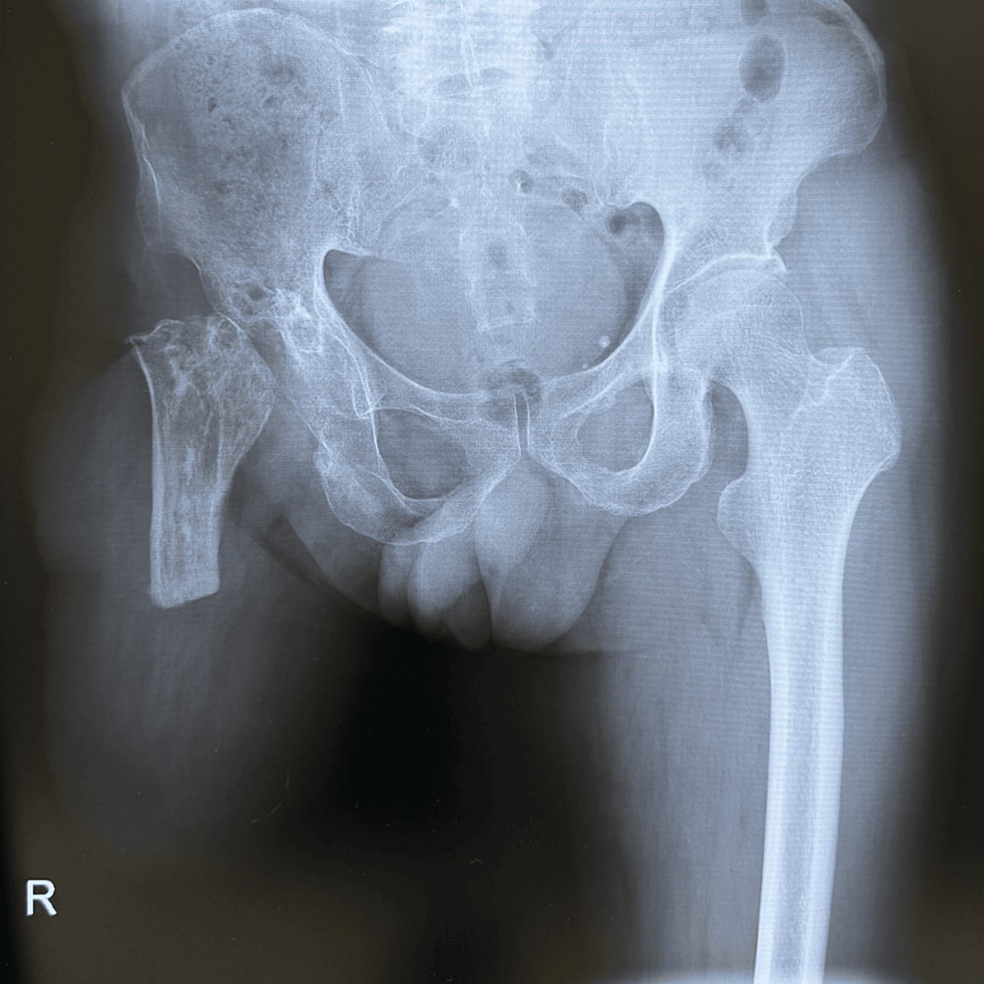
Anteroposterior radiograph of the right Girdlestone hip and transfemoral amputation

The case presented is of a 66-year-old man who sustained a posterior hip dislocation and femoral shaft fracture 40 years ago at the age of 26 due to road traffic trauma. He underwent open reduction of the dislocation and internal fixation of the femoral shaft fracture with a Kuntscher nail. Subsequently, he underwent bipolar hip arthroplasty for necrosis of the femoral head. However, the bipolar hip prosthesis became infected and was removed, leaving the patient in a post-Girdlestone condition. Despite this, the patient was still able to walk with a cane. The femoral shaft fracture had achieved bony fusion but was refractured after a fall, and the Kuntscher nail was removed and replaced with plate fixation. Subsequently, an infection occurred at the same site, leading to the development of chronic osteomyelitis in the femoral shaft. Despite this condition, the patient remained ambulatory using crutches to bear weight on the affected limb. However, at 65, he suffered another fall, refracturing the same area, which rendered him unable to walk. Owing to osteomyelitis, preservation of the affected limb was deemed unfeasible, and the patient underwent a transfemoral amputation (Figure 1).

One month after the transfemoral amputation, the patient was admitted to our convalescent rehabilitation ward for prosthetic rehabilitation. The patient had a history of emphysema as a comorbidity at the time of admission, but it was well controlled with inhaled medications. He presented no other medical complications.

Upon admission, we measured the length from the ischial tuberosity to the stump's end, noting it to be 13.5 cm. The hip joint range of motion was 90 degrees of flexion, -15 degrees of extension, and 20 degrees of abduction. Given the hip's 15-degree flexion contracture, the initial flexion angle of the transfemoral prosthesis was set at 20 degrees.

**Figure 2:**
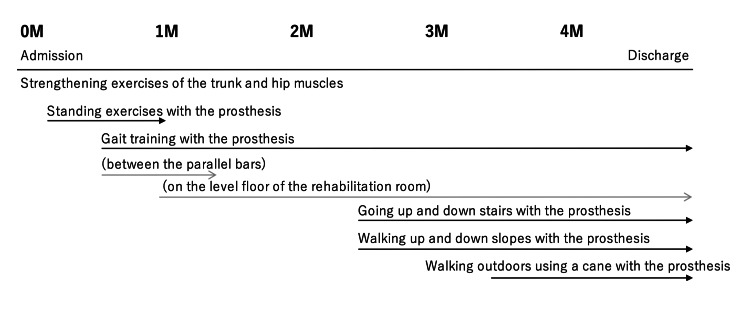
Progress in prosthetic rehabilitation M, months

The progress of his prosthetic rehabilitation is shown in Figure 2.

After his admission to the rehabilitation unit, he began strength training for his trunk and hip extensor muscles. Soon after his admission, a prosthetist made a prosthetic limb, and he began standing and gait training while wearing it. His gait training was initially performed on the parallel bars, and after one month of hospitalization, it was performed on the level floor of the rehabilitation room. After 2.5 months of hospitalization, he began practicing going up and down stairs and walking up and down hills. After 3.5 months, he began outdoor walking exercises and was discharged to his home when his gait function had sufficiently improved (Video 1, Video 2).

**Video 1:** Front view of walking appearance at discharge

**Video 2:** Side view of walking appearance at discharge

**Figure 3:**
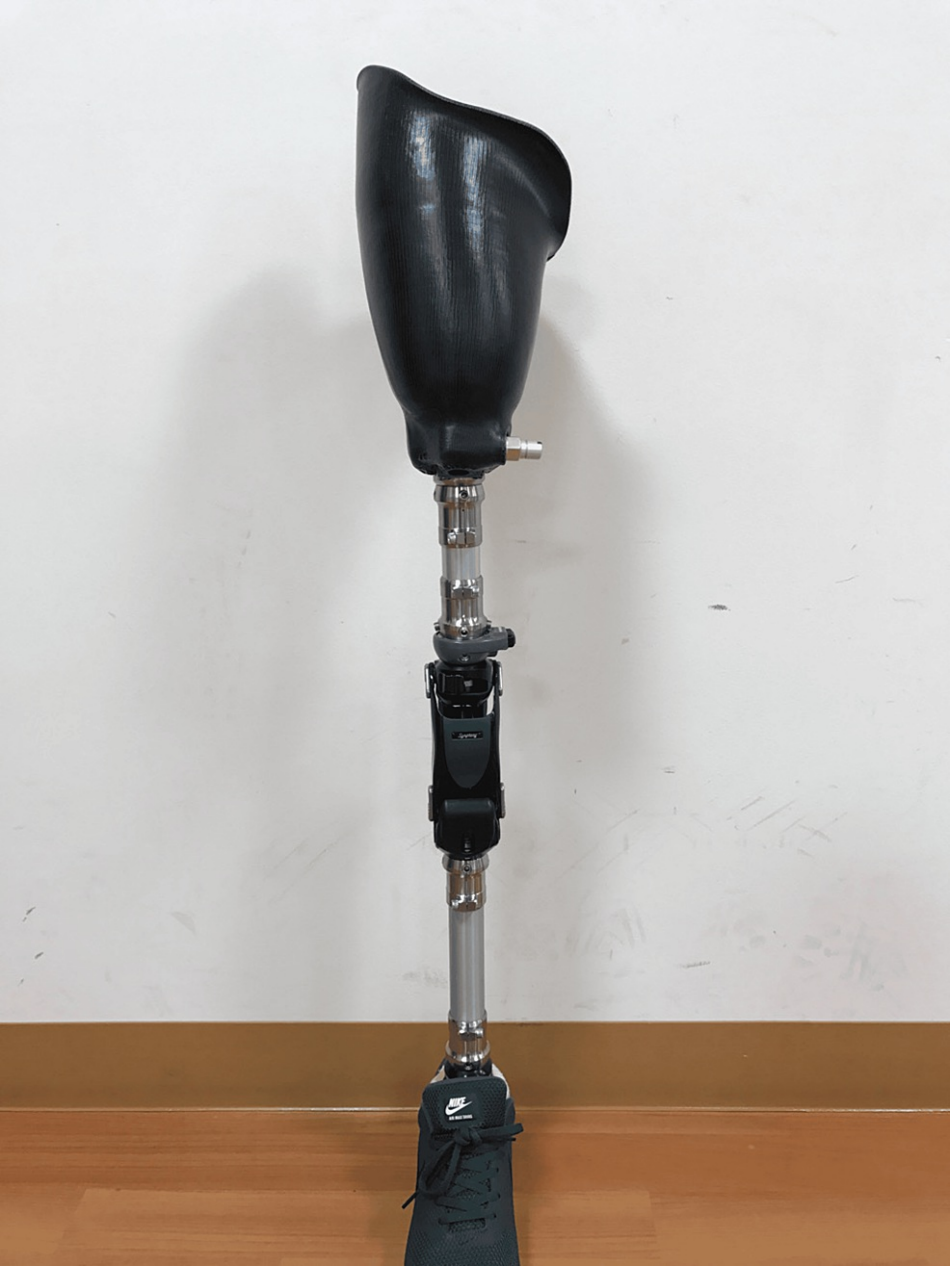
The transfemoral prosthesis that we provided to the patient

The prosthesis we provided to the patient consisted of a quadrilateral socket, pin-lock suspension system, NK-6+L (Nabtesco Corporation, Japan) [7] polycentric knee joint, and 1C30 Trias (Ottobock, Duderstadt, Germany) [8] carbon foot (Figure 3).

At discharge, the 10-meter walk test was 0.93 m/s, the timed up-and-go test was 13.8 seconds, and the 6-minute walk test was 400 meters. The motor functional independence measure (FIM) was 68 points on admission and 85 points on discharge.

We followed this patient for two years. At that time, he was still able to walk with the prosthesis, similar to his capability at hospital discharge. Additionally, he resumed fishing, his hobby, indicating an improved quality of life.

## Discussion

This case report demonstrates that walking with a transfemoral prosthesis is possible after ipsilateral transfemoral amputation following the Girdlestone procedure.

The reason patients were able to walk without pain with the transfemoral prosthesis in this condition is believed to be that there was no significant load on the hip joint while the prosthesis was in place. In a study that created and analyzed a finite element model from computed tomography for transfemoral amputees, the load during prosthesis fitting was concentrated in the ischial region (364 Kilopascal (kPa)) [9]. This means that with a transfemoral prosthesis, the greater load is placed on the ischial bone on the amputated side, not on the patient's hip joint. The Girdlestone procedure preserves the ischial bone. Therefore, the patient was able to walk with the transfemoral prosthesis.

The configuration of the prosthetic limb is not significantly different from that of typical transfemoral prostheses. In our opinion, the shape of the socket of the ipsilateral transfemoral prosthesis after the Girdlestone procedure should be quadrilateral to definitely support the weight maximally on the ischial bone. It is unknown whether the same result would occur with an ischial-ramus containment (IRC) socket, another common socket design, as we have no experience with it.

Manual locking knees have a lower risk of falls than mechanical polycentric knees [10]. Therefore, manual locking knees are preferred in safety-conscious elderly patients [11]. In this patient, a manual locking knee was considered due to poor condition; however, after trialing a mechanical polycentric knee during rehabilitation, it proved usable and was subsequently chosen.

This patient was able to walk, albeit slowly, with the post-Girdlestone hip prior to transfemoral amputation. If the patient was unable to walk due to pain from the Girdlestone hip before transfemoral amputation, it would be expected that the patient would remain non-ambulatory even after the amputation.

This patient has currently been followed up to two years postoperatively, but further follow-up is needed to determine if the patient will be able to walk with a prosthetic limb for a longer period of five or 10 years.

This paper reports what has been done under the Japanese healthcare system. The length of hospital stay and rehabilitation programs may need to be modified depending on the country's system.

## Conclusions

Even after an ipsilateral transfemoral amputation following the Girdlestone procedure, patients can still develop a prosthetic gait. Weight bearing in a transfemoral prosthesis is primarily provided by the ischial bone, not the hip joint. Therefore, with the ischial bone providing primary support, patients can use a transfemoral prosthesis after the Girdlestone procedure. The socket of the ipsilateral transfemoral prosthesis should be quadrilateral to optimize weight distribution on the ischial bone. The knee joint of the transfemoral prosthesis can utilize a mechanical polycentric knee to enhance mobility over locking knee joints. However, prosthetic rehabilitation must be individually tailored to each patient to effectively enable ambulation.
